# From Disease to Illness: Reframing Periodontitis Through an Anthropological Lens

**DOI:** 10.1111/jre.70051

**Published:** 2025-11-07

**Authors:** Carlo Galli, Chiara Moretti, Elena Calciolari, Nikolaos Donos

**Affiliations:** ^1^ Department of Medicine and Surgery University of Parma Parma Italy; ^2^ Department of Social Sciences University of Napoli Napoli Italy; ^3^ Department of Medicine and Surgery, Dental School University of Parma Parma Italy; ^4^ Centre for Oral Clinical Research, Institute of Dentistry, Faculty of Medicine and Dentistry Queen Mary University of London London UK

**Keywords:** cultural awareness, disease and illness, medical anthropology, oral health, periodontal disease, social determinants

## Abstract

While periodontitis is globally recognized as a significant public health problem, its common definition as a plaque‐based inflammatory condition is incomplete. Disease progression, personal experience, and treatment are shaped by social, economic, and structural forces largely invisible in clinical practice and policy. A lens from medical anthropology helps us see periodontitis as more than a clinical diagnosis; it is a lived experience, deeply entangled with a person's social world. The physical reality of inflammation translates into profound emotional distress—from the shame and stigma of bleeding gums and gingival recession to the tangible fear of tooth loss. This personal suffering is often intensified by a societal focus on individual blame, which masks systemic barriers like poor insurance coverage and the simple lack of local care. Ultimately, the cultural language and assumptions surrounding oral health—what anthropologists term explanatory models and semantic networks—powerfully influence everything from a patient's decisions to the public's perception of the disease itself. We argue for a more culturally attuned approach to periodontal health—one that prioritizes prevention, centers the patient's lived experience, and confronts the systemic roots of oral health inequities. By integrating the insights of anthropology with the science of periodontics, we believe we can build a more complete model of care that leads to equitable health outcomes, creating policies and practices that acknowledge both microbial causes and patients' lived realities.

## Graphical abstract

Periodontitis is at once a disease, an illness, and a sickness—where biological pathology, lived experience, and social meaning intersect. The proposed integrated model links upstream structural actions with downstream clinical and educational practices through feedback loops that promote equitable and effective prevention and care.

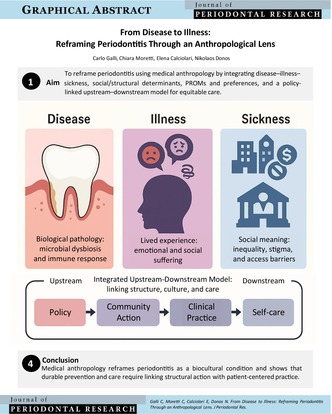

## 1 Beyond the Gums: Why Periodontitis Demands a Broader Lens

### 1.1 Scale and Cost: The Global Footprint of Periodontitis

Periodontal diseases remain a pressing public health concern worldwide [1, 2]. They encompass a spectrum of conditions [3] that affect the tooth‐supporting structures, ranging from reversible gingivitis [4] to periodontitis [5]—an advanced state of chronic inflammation and alveolar bone resorption [6] that eventually leads to tooth loss [7]. Early scientific models of periodontitis focused on the nonspecific plaque hypothesis, which posited that sheer plaque accumulation triggered inflammation [8]. Subsequent refinements identified specific pathogenic species, notably *Porphyromonas gingivalis* , *Tannerella forsythia* , and *Treponema denticola* , as pivotal drivers of disease [9]. More recently, Hajishengallis's polymicrobial synergy and dysbiosis (PSD) model has become the leading framework, portraying periodontitis as a microbial imbalance in which keystone pathogens manipulate the host immune response and enable commensal bacteria to form pathogenic biofilms [10]. Newer studies further suggest that ordinary fluctuations in host–biofilm dynamics alone can produce the heterogeneous tissue breakdown observed clinically [11]. A defining feature of periodontitis is its paradoxical nature: tissue destruction is driven mainly by the host's immune response rather than by direct bacterial action [12]. Persistent, non‐resolving inflammation damages the tooth‐supporting structures, leading to bone loss and, if untreated, tooth loss [13].

Periodontitis is now recognized as one of the most prevalent chronic diseases [14]. A systematic review spanning studies from 2011 to 2020 estimates that about 62% of dentate adults worldwide have periodontitis, with nearly 24% experiencing severe forms [15]. According to the most recent Global Burden of Disease 2021 study, the global age‐standardized prevalence of severe forms is 12.5%. By 2050, severe periodontitis is expected to be the 30th most impactful Level 4 disease/condition, moving up one position in the global ranking since 2021 [16]. These figures underscore an urgent need to strengthen preventive strategies and treatment protocols [17]. Although bacterial and inflammatory mechanisms have been extensively characterized [18, 19], classifying periodontal diseases remains challenging [20]. Different diagnostic thresholds—such as those set by the Center for Disease Control and American Academy of Periodontology (AAP)—produce varying prevalence estimates [21]. Moreover, studies applying the 2017 AAP/EFP classification [22] suggest that up to 72% of adults could be considered periodontitis cases, raising concerns about possible overdiagnosis and the feasibility of adhering to current treatment guidelines [23]. These evolving definitions affect clinical planning, resource allocation, and the economic sustainability of care [24]. Periodontitis also imposes heavy macro‐economic costs [25]. A 2018 cross‐national analysis estimated total costs at about €156 billion for 32 European countries and about €150 billion for the United States, of which roughly 80% were indirect losses tied to absenteeism and disability [26]. These figures still understate the full burden: recent modeling assigns substantial *intangible* costs to periodontitis, capturing pain, dietary limitation, lost self‐confidence and social anxiety that never appear on balance sheets [24].

Periodontitis is increasingly attracting the same analytic toolkit used for other chronic diseases, with cost‐effectiveness, cost‐utility and cost–benefit analyses beginning to guide resource allocation [27, 28, 29, 30]. Recently, the Economist Intelligence Unit (EIU) report highlighted the economic and social costs related to periodontitis and demonstrated the economic benefit of increased prevention, early diagnosis and management of this disease [31]. The recognized role of periodontitis as a risk factor for systemic conditions (e.g., cardiovascular disease, diabetes, respiratory infections) heightens these costs further by complicating overall health management [32]. In Switzerland, a simulation by Ramseier et al. [33] projected that accurate diagnosis and professional periodontal care could save up to 7 billion francs in out‐of‐pocket and social welfare expenses. Additional data from Saleh et al. [34] emphasize how untreated periodontitis, when intertwined with chronic diseases, inflates overall medical expenditures, although modeling in the same review suggests that regular supportive periodontal care costs only about €29 for every extra tooth‐retention year, a fraction of the downstream expenses otherwise incurred. The potential for cost savings through early periodontal treatment and prevention is substantial and can reach several thousand dollars per capita [35], as timely intervention reduces complications that would otherwise require more complex and expensive medical treatments [36]. Yet these numbers tell only half the story; they do not explain why the burden falls unevenly across populations.

Building on this evidence base, the present paper sets out to move beyond a strictly biomedical account by tracing how economic pressures, structural inequities, and cultural meanings converge to shape the course of periodontitis, and by proposing an anthropologically informed biopsychosocial framework to guide future research, clinical decision‐making, and policy.

### 1.2 Beyond Biology: Unmasking Structural and Cultural Drivers

Numbers alone cannot fully capture the social, structural, and cultural engines that drive periodontal risk [37]. Despite growing recognition of the costs of periodontitis on health systems, implementing preventive and maintenance care on a large scale remains difficult [17]. For example, in Norway, meeting European guidelines for periodontal maintenance could consume approximately 71% of all clinical hours allocated to adult care [23], posing untenable financial and logistical demands. This tension calls for a sustainable approach that balances public health goals with real‐world resources [38, 39]. Pathogenesis, prevalence and cost justify clinical urgency, but they often cannot, by themselves, explain *who* develops advanced disease or *why* some communities remain chronically underserved [37, 40]. A further analytical step is required; a key element is the widespread lack of awareness [41]. Across settings, understanding of periodontitis—its course, key risk factors such as tobacco use or diabetes, likely consequences, and preventive measures—remains limited in the general public and among patients, with particularly marked gaps in some groups [42]. Such limited awareness undermines timely help‐seeking and weakens opportunities for early prevention on a population scale.

Although clinical and economic hurdles are well documented, they also reveal deeper sociocultural and structural inequities [43]—a dimension that medical anthropology is in a unique position to address [44, 45]. Concepts from anthropological studies, particularly “disease,” “illness,” and “sickness,” offer a lens to view periodontitis not merely as a clinical entity but as an embodied experience shaped by broader social forces [46, 47]. Socioeconomic disparities, cultural beliefs about oral health, and systemic barriers to care combine with biological and medical factors, ultimately determining who benefits from timely interventions and who endures disease progression [48]. For instance, while some cultural contexts treat routine dental checkups and scaling as indispensable, others may regard them as optional or prohibitively expensive due to a lack of insurance or limited access to dental services [49]. The integration of anthropological insights with current periodontal research permits a more comprehensive examination of the biological, behavioral, and social forces that shape periodontal health outcomes.

## 2 Culture, Power, and Plaque: An Anthropological View

Medical anthropology examines how societies interpret disease, choose therapies, and negotiate the meanings of health conditions [50, 51]. Instead of confining health and disease to purely organic phenomena, this perspective situates them within broader social, historical, economic, and political contexts [52]. Its central premise challenges Cartesian dualism separating mind and body and recognizes that “the mindful body” [53] arises from the intersection of individual experience, cultural metaphors, and broader social and power structures [54]. In dentistry, anthropological insights can contribute to understanding why oral health disparities persist and how cultural meanings attached to the mouth and teeth influence perceptions of periodontitis [55]. As studies repeatedly show, the onset and progression of periodontitis depend on more than bacterial etiology [56]. Socioeconomic status, cultural diet preferences, and access to preventive services all modulate whether microbial plaque accumulation evolves into frank disease [37, 57, 58].

Broadly speaking, Paul Farmer's famous concept of structural violence has helped reveal how systemic economic and political processes systematically disadvantage certain populations [59, 60, 61]. Here “structural violence” means hidden harm from social and economic arrangements—unequal access to care, education, or housing—that quietly constrain health and well‐being. It refers to patterned inequities built into institutions and policies, not necessarily to overt acts of physical aggression. Periodontitis frequently illustrates this disadvantage: lower‐income groups experience higher rates of advanced disease due to limited access to preventive services and perceived cost barriers [62]. In the U.S. and in Europe, racialized minorities with low income face the greatest disparities in dental care access [37, 63]. Privatized dental care systems compound these disparities [64]. Countries like the U.S., where dental care is largely privatized, exhibit the highest inequalities in self‐rated oral health—with a 40.4% absolute difference between the most and least educated groups [65]. Insurance systems in many high‐income countries classify advanced periodontal procedures as “elective,” excluding them from coverage or imposing substantial co‐pays [66].

Structural barriers also extend beyond cost: rural populations, as seen in China, encounter reduced access to dental services compared to urban counterparts, exacerbating untreated disease and poor oral health‐related quality of life [67, 68]. Low socioeconomic status (SES) populations are disproportionately affected by structural constraints—such as limited time off, income inequality, and unequal access to affordable nutrition—that reduce opportunities for preventive dental care and healthy dietary choices [69]. In line with Raittio et al., even if best‐practice guidelines exist, they become nearly impossible to implement at scale [23].

Equally important is the stigma often attached to gum disease [70]. Public health campaigns commonly emphasize personal responsibility for hygiene, potentially implying that afflicted individuals are at fault [71]. This, however, can discourage early visits and sustain a cycle of denial or fear, wherein patients avoid checkups until pain or tooth loss demands care [58]. Anthropological approaches and analysis thus probe how resources, beliefs, and power imbalances shape oral health behaviors, going beyond conventional frameworks, helping us look upstream of “nonadherence” discussions, by adding context around patients' social and economic challenges [72]. For instance, oral health disparities disproportionately affect Hispanic and Native American populations in Texas, where the lack of affordable dental insurance prompts many to forgo essential treatment, reinforcing cycles of poor oral health and broader systemic inequality [73].

These structural barriers—cost, limited coverage, social stigma, logistical constraints—signal the need for culturally informed and community‐based strategies. Encouraging earlier detection and collaborative care can help prevent advanced disease and tooth loss [74]. At the same time, public policy must confront the systemic underfunding of dental services and develop coverage structures that promote timely preventive care [75]. When structural violence is considered alongside microbial dysbiosis and plaque control, clinicians, policymakers, and community stakeholders are better positioned to design responses that reflect the full complexity of periodontal disease. The next section drills down to the level of personal experience, showing how people *live* these structural forces through the intertwined dimensions of disease, illness and sickness.

## 3 Disease × Illness × Sickness: Mapping the Lived Mouth

**FIGURE 1:**
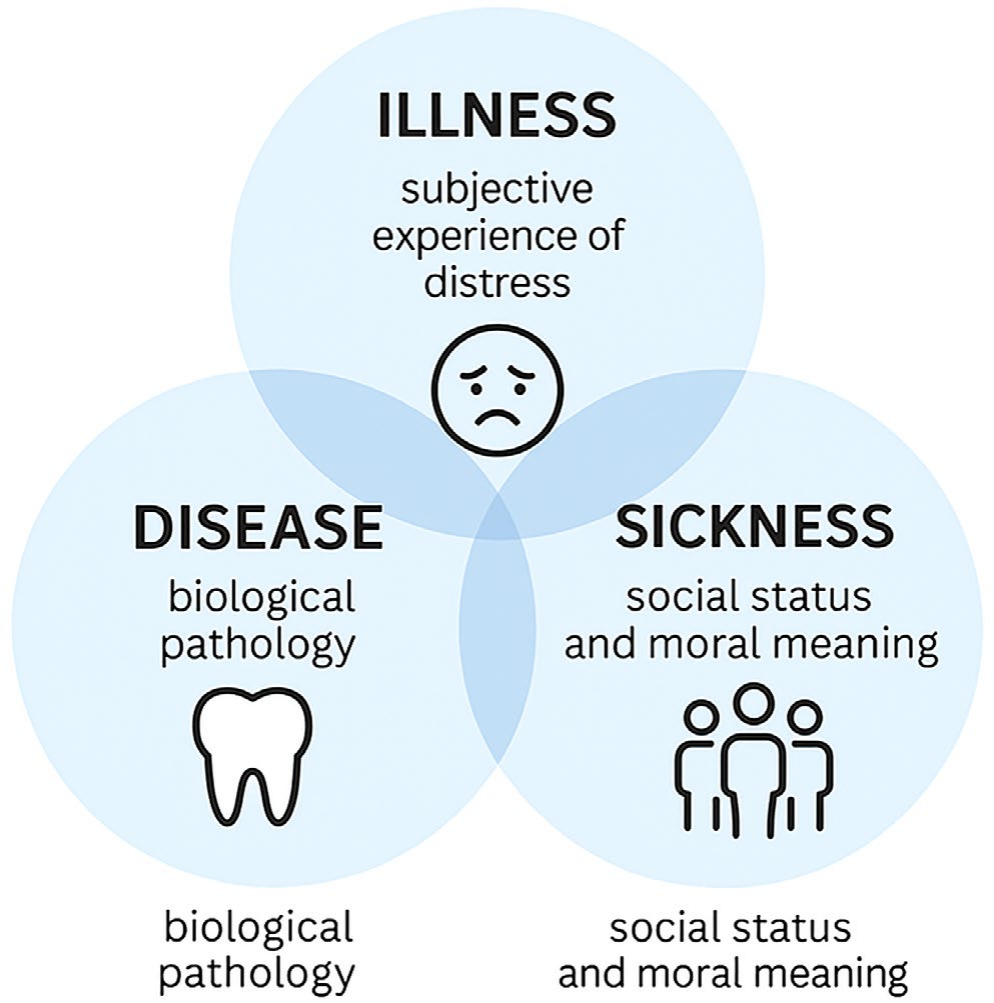
The tripartite model of pathology proposed by Eisenberg and Kleinman.

Following Eisenberg and Kleinman, we can distinguish disease (biological pathology), illness (the felt, subjective experience of distress) and sickness (the social status and moral meaning assigned to being unwell) [46]. These three lenses, when viewed side by side (Figure 1), prevent the biomedical description of pathology from eclipsing what patients feel or how society judges them [46]. Though these terms are often conflated in everyday usage, their differences reveal important dimensions of how periodontitis is perceived, experienced, and addressed. Importantly, they also highlight how, generally, individuals construct meaning around pathology and care.

Within biomedicine, the western current medical system and approach that focus on the biological dimension of pathology, disease is conceptualized as a pathological state identifiable through objective criteria [76]. Periodontitis is for instance defined by measurable biological markers, including clinical attachment loss (CAL), the presence of periodontal pockets, alveolar bone resorption, and inflammatory biomarkers [3, 77, 78]. Recent research has significantly expanded our understanding of periodontitis beyond a simple bacterial infection [79], highlighting its multifactorial etiology [80, 81] involving host–microbiome interactions [82], immune dysregulation [83], and systemic implications that can contribute to the pathogenesis of atherosclerosis, insulin resistance, and neuroinflammation [84, 85, 86, 87]. Periodontitis is biologically complex; most research understandably focuses on its many ramifications. Yet, even this essential body of knowledge still misses the patient's lived reality. Even the most sophisticated pathophysiological models can unintentionally sideline patients' daily experiences.

Kleinman contended that biology only constituted a facet, albeit undoubtedly important, of the complex phenomenon of pathology, and biomedicine would often not fully capture the patients' own experience, that is, *illness*. Kleinman's use of the term “illness” captures the subjective lived experience of suffering—shaped by cultural frameworks, social relationships, and individual history [51]. The idea of illness encompasses how the disease is felt in daily life—how symptoms alter or disrupt routines, self‐image, or social relations [88]. Consequently, disease and illness are independent concepts: it is possible to have a disease and not be aware of it (i.e., no illness), and it is possible to have an illness (i.e., a perceived discomfort) in the absence of a clinically detectable disease [89, 90]. Periodontitis is often described as a silent disease due to its gradual and initially painless progression, leading many patients to be unaware of early symptoms. However, as the condition advances, changes in the periodontium and teeth can evoke considerable emotional distress [91]. Additionally, these morphological alterations, namely gum recession, tooth migration and tooth loss may become permanent, functioning much like “scars” that visually and physically mark the progression of periodontitis [92] and serve as ongoing reminders of how one's body has been irrevocably affected by the disease. Such embodied signs can reshape a patient's sense of identity, reinforcing the emotional weight of the illness by continuously reminding them of past suffering and potential future deterioration [93, 94].

Awareness of disease and its consequences links these three dimensions. When people lack a basic grasp of how periodontitis develops, which behaviors and conditions elevate risk, what early signs signify, and how prevention works, their illness experience is shaped by uncertainty and delay, and the social labeling of sickness can slide toward stigma rather than action [95]. Recent insights confirm that many patients encounter psychosocial challenges such as shame about the condition, fear of tooth loss, and social isolation [96]. They may conceal their mouths when talking or avoid certain social settings [70, 97]. A recent multicenter study confirmed that periodontitis is associated with markedly worse Oral Health Impact Profile‐14 (OHIP‐14) scores than a healthy periodontium, even after adjusting for age, smoking, BMI and ethnicity, underscoring the psychosocial weight of the disease [98]. Studies that employed diaries or in‐depth interviews [99] reveal that many of those who experience progressive periodontitis engage in complex workarounds to hide signs of disease (e.g., not smiling, chewing gum or mints to mask malodor), which significantly disrupt social interactions. An anxious sense of losing control, guilt over poor self‐care, and dread of future tooth loss further intensify the illness dimension [100]. Therefore, researchers and practitioners have become more aware that beyond measuring objective clinical endpoints like pocket depth or attachment levels, the inclusion of patient‐centered measures—for example, Oral Health Quality of Life (OHRQoL) scales—and more exploratory, open‐ended qualitative approaches (diaries, semi‐structured interviews) can significantly improve our understanding of the burden of periodontitis on patients [101].

This not only reflects the personal dimension—illness as subjective experience—but also resonates with the sense of social threat that anthropologists call *sickness*, meaning the outward social definition of having a disease that may be stigmatizing or shameful. Sickness describes the social definition of being unwell—how society views and reacts to those deemed “sick” [74]. Moreover, the concept of sickness comprehends the socialization and shaping of both disease and illness [102]. Sickness emerges as the final result of micro‐ and macro‐level historical, economic, and political processes that generate power asymmetries and inequitable social relationships, ultimately impacting individuals' lives and health status. In other words, sickness emphasizes the sociopolitical construction of what it means to be unwell, as signs and symptoms become interpreted and labeled as disease within specific historical and sociocultural contexts [103, 104].

Recent qualitative research underscores precisely these multiple layers of illness and sickness. In diaries and semi‐structured interviews with referred periodontitis patients, participants portrayed a sense of stigma, “concealment”, guilt, anxiety, and social disruption that shaped their disease experience. These studies also highlight the crucial role of the patient–dentist relationship, where receptivity, communication style, and supportive clinical environments can help alleviate anxiety and emotional distress [105]. Many patients have reported that, after adequate supportive communication with clinicians and completion of nonsurgical periodontal therapy, they underwent a positive shift—feeling relief, pride, and reengagement in social life [106]. These psychosocial transformations could be placed under Locker's (1988) conceptual model of oral health, illustrating how intangible emotional burdens ultimately weigh on the patient's wellbeing as much as the pocket depths or the alveolar bone levels so central to the biomedical perspective [107].

Thus, periodontitis is more than a biological disease; it is an evolving narrative shaped by personal biography, social processes, and relationships, leading either to deeper isolation and worry or, with care, to renewed self‐esteem and control. Recognizing how diverse cultural codes and expectations define “ideal” dental health clarifies why people differ in their views of prevention, treatment, and oral well‐being [88]. Ultimately, distinguishing disease, illness, and sickness helps periodontists see how a clinical entity, such as periodontitis with alveolar bone and ligament loss, intersects with the felt distress of patients and the social frameworks through which they find meaning and place blame [47]. These experiential layers raise a further question: why do dentists and patients often describe the same bleeding‐gums condition so differently? Two classic anthropological tools—explanatory models (EMs) and semantic networks—offer possible answers.

## 4 Explanatory Models and Semantic Networks: Kleinman and Good in Periodontics

### 4.1 Explaining the Unseen

Arthur Kleinman's EM framework has long been a cornerstone of medical anthropology, offering a structured approach to understanding how patients, healthcare providers, and communities conceptualize health and disease [108]. Kleinman argued that diseases are never just biomedical phenomena but are also cultural, social, and psychological constructs that influence how individuals perceive, experience, and respond to illness [109]. At the heart of his framework is the premise that an illness episode is shaped by competing EMs—those held by the patient, the family, and healthcare professionals (Table 1). These models seek to answer fundamental questions about the nature, causes, treatments, duration, and severity of a disease; as such, health beliefs inevitably become culturally constructed interpretations rather than purely biomedical facts [110].

**TABLE 1:** Two conflicting explanatory models for periodontal disease.

Clinician's biomedical model	Patient's alternative model
Chronic inflammatory condition caused by bacterial dysbiosis	Gum bleeding seen as minor or normal; tooth loss viewed as part of aging
Focus on professional debridement, antimicrobial therapy, and surgery	Preference for herbal rinses, home remedies, or folk practices
Recommend daily brushing and interdental cleaning and schedule maintenance visits (supportive periodontal care)	Flossing seen as trivial or unnecessary if there is no pain
Bacterial plaque identified as the root problem, requiring strict control	Social/financial realities (low income, minimal insurance) override preventive priorities

Kleinman's concept of EMs shows how identical clinical signs can yield divergent understandings. A dentist may view periodontitis as a chronic inflammatory disease caused by bacterial dysbiosis, while a patient might regard gingival bleeding as a minor nuisance or see tooth loss as an unavoidable part of aging. In marginalized communities where resources are scarce, structural barriers—such as limited insurance or geographical isolation—often intensify these differing views, and this shapes how early symptoms are interpreted or even ignored [111]. Such mismatches in EMs can undermine care [112]. For example, if a patient relies on herbal mouth rinses and the dentist fails to ask about—or dismisses—these practices, the patient may feel unheard or disrespected and disengage from treatment [113]. This dismissal not only undermines trust but also reflects a broader epistemic asymmetry that excludes the patient's experiential knowledge [114]. Kleinman's notion of *explanatory model negotiation* therefore advocates integrating the patient's perspective to build trust and encourage adherence, obviously without losing sight of the current knowledge about that specific clinical condition, but recognizing that the patients' lived, culturally mediated expertise can offer critical insights into effective care strategies [115]. It has been convincingly shown that standardized health messages may fail if they do not align with local beliefs [116]: communities where tooth loss is widely accepted, or where recommendations to daily flossing are seen as trivial, require educational strategies that first address these normalized attitudes [117]. Building on this point, we distinguish communication gaps—when essential information never truly reaches or is not actionable for its intended audience—from miscommunication, when information does reach people but clashes with their EMs. Effective periodontal education must address both through sustained, co‐created, culturally and linguistically adapted messaging [115].

In line with Tullio Seppilli's notion of *cultural calibration*, this means adapting and tailoring medical discourse and practice to the patient's worldview and illness lived experiences [118]. Doing so involves understanding the values, meanings, and cultural frames of reference that inform people's lifestyles, beliefs, and motivations in seeking care [118]. As highlighted in the EIU report, societal‐level prevention is crucial for the prevention of periodontitis, especially as it is a disease prevalent in deprived areas [31]. Interventions to promote better periodontal health need to be embedded into relevant and targeted community settings [119, 120]. Taking these factors into account may allow providers to overcome barriers of mistrust, cognitive dissonance, and divergent expectations, fostering more effective and respectful healthcare interactions [121, 122]. As highlighted by Moretti [114], the knowledge that patients acquire through firsthand experience of illness often remains overshadowed by purely biomedical approaches, which often overlook the patients' adaptive strategies, which are developed through prolonged engagement with their condition, and which usually address multidimensional factors (e.g., emotional, social components). Integrating patient‐derived insights not only improves adherence but also enables a transdisciplinary model blending professional expertise with patients' own understanding and coping strategies [123]. Recognizing the patient as a valuable “expert” in their own condition—not in the pathological process per se but in what they are experiencing—also means shifting from a model where patients are simply instructed to comply, toward one of shared decision‐making [124, 125]. Providers and patients can *compete*, as in Latin “cum‐petere”—literally “go forward together”—by creating a shared frame of reference that respects both scientific evidence and the patient's lived reality [126]. When healthcare professionals validate patients' beliefs and experiences, they can gain deeper insights into social and emotional factors that may be affecting treatment outcomes [127]. Such an approach reduces the risk of alienating patients and helps co‐construct interventions that are not only clinically sound but also culturally and personally meaningful [123, 128]. Enlisting cultural brokers who can bridge biomedical information and community values can enhance acceptance of periodontal care, fostering a more equitable and patient‐centered approach [129]. Table 1 illustrates how each side may conceptualize periodontitis differently. Without reconciling these models, effective communication, patient adherence, and sustainable care may be compromised.

### 4.2 Speaking in Symbols

Byron Good's concept of “semantic networks” complements Kleinman's emphasis on EMs by highlighting the central role of language in shaping how people understand and respond to illness [130, 131]. Good challenges the view that diseases are merely collections of objective symptoms, arguing instead that language and discourse actively construct the social and moral dimensions of pathology [132]. In the case of periodontitis, it could be assumed that the terms dentists use to describe “severe” periodontitis may appear technically precise, but patients can interpret them as moral judgments (“You haven't taken care of yourself”), social threats (“People will think I'm unhygienic”), or personal failings.

Medical language, therefore, does not merely label clinical states; it also embeds them within broader cultural, ethical, and emotional contexts [133]. Dental consultations, public health campaigns, and even casual conversations about oral hygiene all become sites where meanings are formed, negotiated, challenged, or reinforced. These overlapping layers of meaning help explain why patients might prioritize esthetic or psychosocial dimensions—like embarrassment at work or fear of aging—over measurable indicators such as pocket depth or attachment loss [134].

In a seminal study of “heart distress” in Iran, Good showed that a single illness term could fuse palpitations, financial strain, and moral judgment into one felt syndrome—a semantic network of symbols and symptoms [131]. A similar lattice of meaning surrounds “gum disease,” where plaque‐related pathology mingles with fears of shame, aging, and neglect. Yet biomedical terminology often diverges from patients' views [135]: they may downplay its seriousness or fail to recognize it as an illness [136]. Good argues that analyzing such semantic networks requires moving beyond differential diagnosis to observe how phrases like “gum disease” are used in everyday conversation and which anxieties or moral feelings they awaken. Each node—embarrassment at work, perceived poor hygiene, fear of aging, neglect, stigma, pain, or costly treatment—can cluster with “gum disease,” creating an emotional and social syndrome that may overshadow microbial explanations.

Good's perspective shows that a label's clinical significance arises not only from its link to pathophysiology but also from how it is used in everyday social contexts [131]. Conversations—between a mother and daughter about tooth pain or friends discussing implant costs—shape the meanings of “periodontitis” or “bleeding gums.” Just as Good found with “heart distress” in Iran, which evoked concerns about fertility, pollution, family conflict, and sadness, “periodontitis” can summon experiences ranging from financial shame to moral narratives about cleanliness. These linguistic networks are dynamic, reshaped each time the term is spoken [131].

Good emphasizes that semantic network analysis does not aim to fix a single, universal meaning for a disease label. Instead, it explores how an illness term gains significance by repeatedly linking to situations, feelings, and moral discourses within a community. These evolving connections shape the term's symbolic weight. Applied to periodontitis, a semantic analysis of expressions such as “gum disease” or “periodontitis” would trace how local experiences—financial constraints, social factors, or barriers to care—interact with these terms. Further research is needed to see whether mapping such associations can help predict or improve patient responses to diagnosis and treatment. Even now, this approach clarifies the cultural tensions and influences behind patients' attitudes toward their disease and care.

**FIGURE 2:**
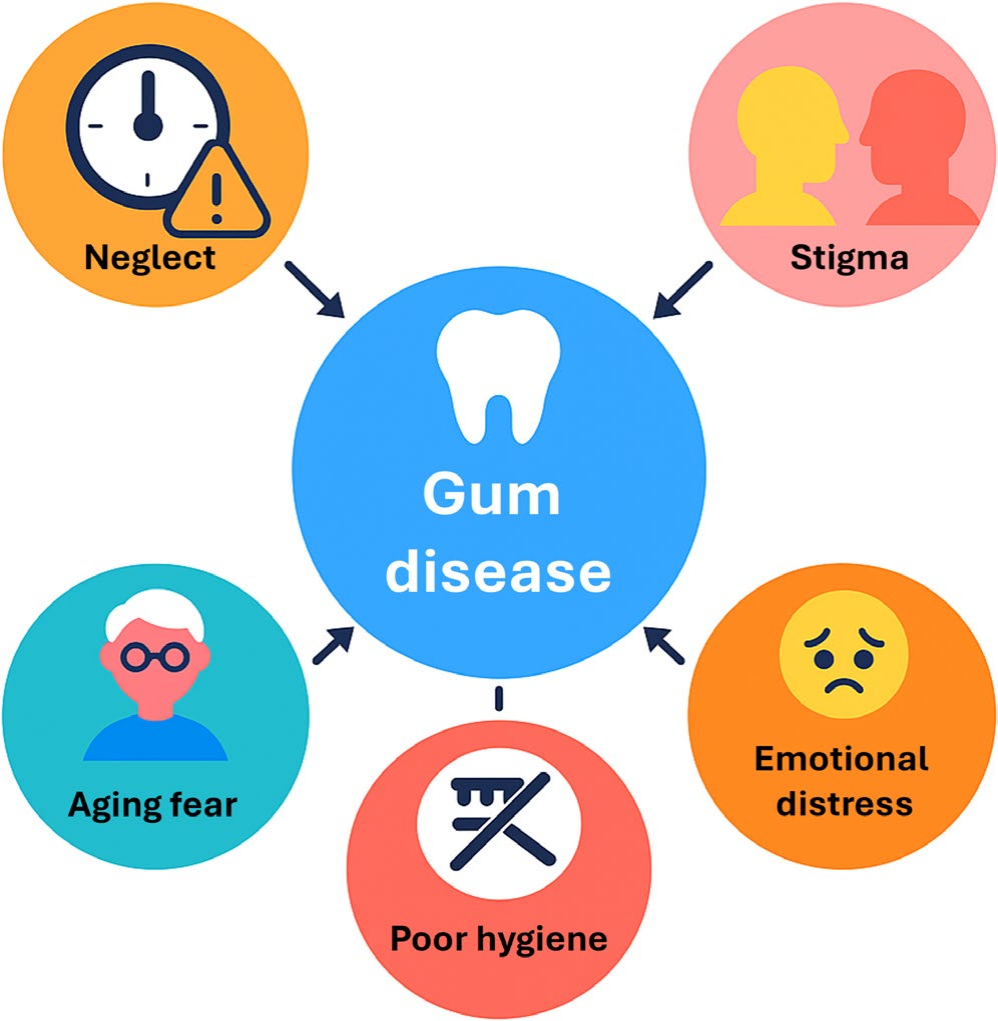
Sample semantic network for “Periodontal Disease.”

Figure 2 shows a hypothetical example of a simple semantic network. Such a network is formed by key concepts associated with the illness—the nodes—and connected by edges, which represent the relations that link the concepts. In this example, a single phrase—“gum disease”—can eventually connect multiple themes such as social judgment, moral responsibility, or fear of aging. Each node reinforces or modifies the patient's overall perception of periodontitis, shaping attitudes toward professional care. In a clinical setting, if a patient hears “gum disease” and immediately associates it with personal neglect or social criticism, they may feel defensive, embarrassed, or hopeless, thus delaying follow‐up visits. These semantic associations do not remain idle: they can push a patient to delay seeking care, or to hide early symptoms to avoid moral condemnation. They can also shape how a patient interacts with a clinician [132].

In line with Good's argument, a purely biomedical explanation of plaque accumulation, microbial dysbiosis, and inflammation, while crucial for understanding mechanisms, may fail to address these layered cultural scripts that attribute moral or emotional significance to gum disease. A single term can evoke many associations—from fear and shame to financial anxiety—so clinicians should acknowledge this “polysemy”, which is precisely what invests an illness term with its emotional force [131].

The two foundational concepts—EMs and semantic networks—can help dental professionals, public health policymakers, and educators understand the conceptual and linguistic barriers that delay care. A single illness idiom can weave together personal, social, and moral meanings that deter patients from seeking formal treatment. If a dentist recognizes that “gingival bleeding” might recall deeply felt moral undertones or anxieties, the clinician can strive to reframe the issue in empathetic ways. Similarly, health campaigns might avoid overly clinical or stigmatizing labels, instead adopting language that acknowledges the patient's lived experiences—cultural orientations, socioeconomic burdens, or generational beliefs. Integrating these anthropological insights into everyday dental practice can reduce miscommunication and foster care environments where patients feel respected and understood [137]. To address the need for further research in this area, empirical studies are needed to explore how EMs and semantic networks influence patients' understanding and management of periodontal disease. This might be a very relevant step to ultimately allow for more tailored and effective care strategies in dentistry.

## 5 From Insight to Chairside: Turning Anthropology into Action

### 5.1 Building Trust in the Operatory

Culturally aware periodontal care calls for open discussions about oral‐hygiene habits, beliefs on gingival bleeding, and use of traditional remedies. Communication improves when clinicians offer a brief first‐visit orientation—covering disease course, risks, early signs, and prevention—using plain language and visuals. They can confirm understanding with a short teach‐back, and align next steps with each patient's priorities and constraints. These steps help close true communication gaps, ensuring that essential information is provided and understood, while also minimizing miscommunication by adapting content to the patient's sociocultural and clinical context [115, 138].

By validating the illness experience rather than dismissing it, clinicians can build trust and improve adherence to treatment regimens [54]. This trust‐building can be bolstered by community outreach programs located in schools, workplaces, or other accessible venues, where periodontal screenings and health workshops address misconceptions about periodontitis [139]. Moreover, partnering with community leaders or organizations helps ensure that programs are co‐created rather than imposed from the top down [57].

### 5.2 Team Science, Community Reach

Meanwhile, in certain cultural contexts, such as rural areas, simply traveling to a dentist for repeated scaling may be unfeasible due to significant geographical and infrastructural barriers [140]. For instance, border regions like those between the U.S. and Mexico—particularly among immigrant populations—face heightened challenges in accessing dental care, with disparities in availability and affordability of services leading to worsened oral health outcomes [141, 142]. Families in “food deserts” may lack realistic options to consume less cariogenic diets or to find time for rigorous oral hygiene while working multiple jobs [143]. Limited access to nutritious food and high rates of childhood dental decay in underserved areas, such as the El Paso border region, highlight the compounding effects of socioeconomic disadvantage on oral health [144]. Strengthening these efforts through interprofessional collaboration is also essential. At the community level, primordial and primary prevention depend on communication strategies that are proactive, repeated, and co‐designed with local stakeholders. Examples include multilingual campaigns highlighting early signs and prevention, integration of smoking‐cessation and diabetes‐control messages into periodontal outreach, and chairside materials adapted for different literacy levels [145]. Embedding such initiatives in schools, workplaces, and primary care and pharmacy settings extends reach and equity, aligning with international calls for population‐level oral‐health action [146].

Periodontists, nutritionists, physicians, cultural anthropologists, and social workers can tackle not only the microbial aspects of periodontal inflammation and the surgical dimensions of treatment, but also the socioeconomic and cultural factors that hinder care [147, 148]. Despite the potential benefits—such as reduced disease progression, improved overall health, and lowered healthcare costs—many Western countries rely on fragmented healthcare infrastructures that make integrated services challenging to implement [55, 149]. Ultimately, addressing periodontitis within a broader public health framework involves advocating for policy changes that improve access to preventive services [150]. Rather than labeling advanced therapies as “elective,” policymakers and healthcare systems should recognize the long‐term savings and quality of life gains that come from early intervention [151].

### 5.3 Listening in Metrics: PROMs for Periodontal Care

While anthropological analyses illuminate the cultural, socioeconomic, and experiential facets of periodontal disease, the growing emphasis on patient‐reported outcome measures (PROMs) provides a practical bridge between these insights and daily clinical practice [152]. Historically, periodontal studies have relied predominantly on clinical or surrogate measures—probing depths, clinical attachment levels, or radiographic bone height [77]. However, such metrics alone may fail to capture the real‐life implications of periodontitis on patients' everyday experiences. As contemporary health paradigms shift toward a holistic, biopsychosocial view, researchers increasingly underscore the value of PROMs to assess aspects like pain, self‐esteem, social confidence, and overall well‐being [153, 154].

A PROM is any measurement that patients themselves report directly, without interpretation by clinicians [155]. In periodontics, PROMs can reveal whether a reduction in probing depth is truly meaningful to individuals—improving their ability to eat, reducing discomfort or sensitivity, or alleviating esthetic concerns [156]. Notably, Tsakos and colleagues showed that a change of ≈5 points on the Oral Impacts on Daily Performances index is the smallest difference patients judge as worthwhile after periodontal therapy, providing a benchmark for clinically meaningful improvement [157]. Including PROMs in clinical trials therefore strengthens the relevance of findings to patients, facilitates shared decision‐making, and enhances patient adherence [158].

Consensus initiatives—such as the Cochrane Oral Health group's core‐outcome sets [159], and the FDI's partnership with the International Consortium for Health Outcomes Measurement [160]—have begun to establish standardized outcomes that combine both biomedical and patient‐centered metrics. While these efforts signal progress, the perspectives of *people with lived experience* (PWLE) [161] of periodontitis are still underrepresented, especially among those from cultural backgrounds with limited access to care [162]. By engaging these patient perspectives from the outset—identifying relevant outcomes, measuring them systematically, and incorporating feedback into clinical guidance—researchers and practitioners can help ensure that findings resonate with real‐world contexts. In addition to PROMs, it is crucial to consider patients' preferences for treatment. Preferences reflect what individuals value most in the choice among available therapies—whether convenience, comfort, esthetics, cost, or treatment duration—and are shaped by their sociocultural background, economic circumstances, and clinical history. While PROMs capture the outcomes patients experience, preferences inform the decisions they are willing to make in the first place. Evidence from chronic disease management confirms that aligning treatments with patient preferences improves adherence and health outcomes, underscoring the importance of eliciting such preferences in clinical practice [163]. Exploring these preferences through open discussion or shared decision‐making ensures that treatment plans integrate both the objective needs of disease control and the subjective realities of illness experience. Incorporating preferences in this way supports a care environment where patients feel respected, understood, and genuinely involved in their periodontal care. Taken together, these approaches remind us that periodontitis is an embodied, sociocultural phenomenon—one that cannot be fully understood or treated by clinical endpoints alone. By aligning biological metrics with PROMs and cultural narratives, periodontics can evolve into a more comprehensive practice that accounts for suffering in all its dimensions. The result is healthcare that is scientifically rigorous, culturally resonant, and truly responsive to patients' lived experiences—ultimately contributing to more equitable and effective periodontal health for all.

### 5.4 From Upstream to Downstream: An Integrated Model for Action

**FIGURE 3:**
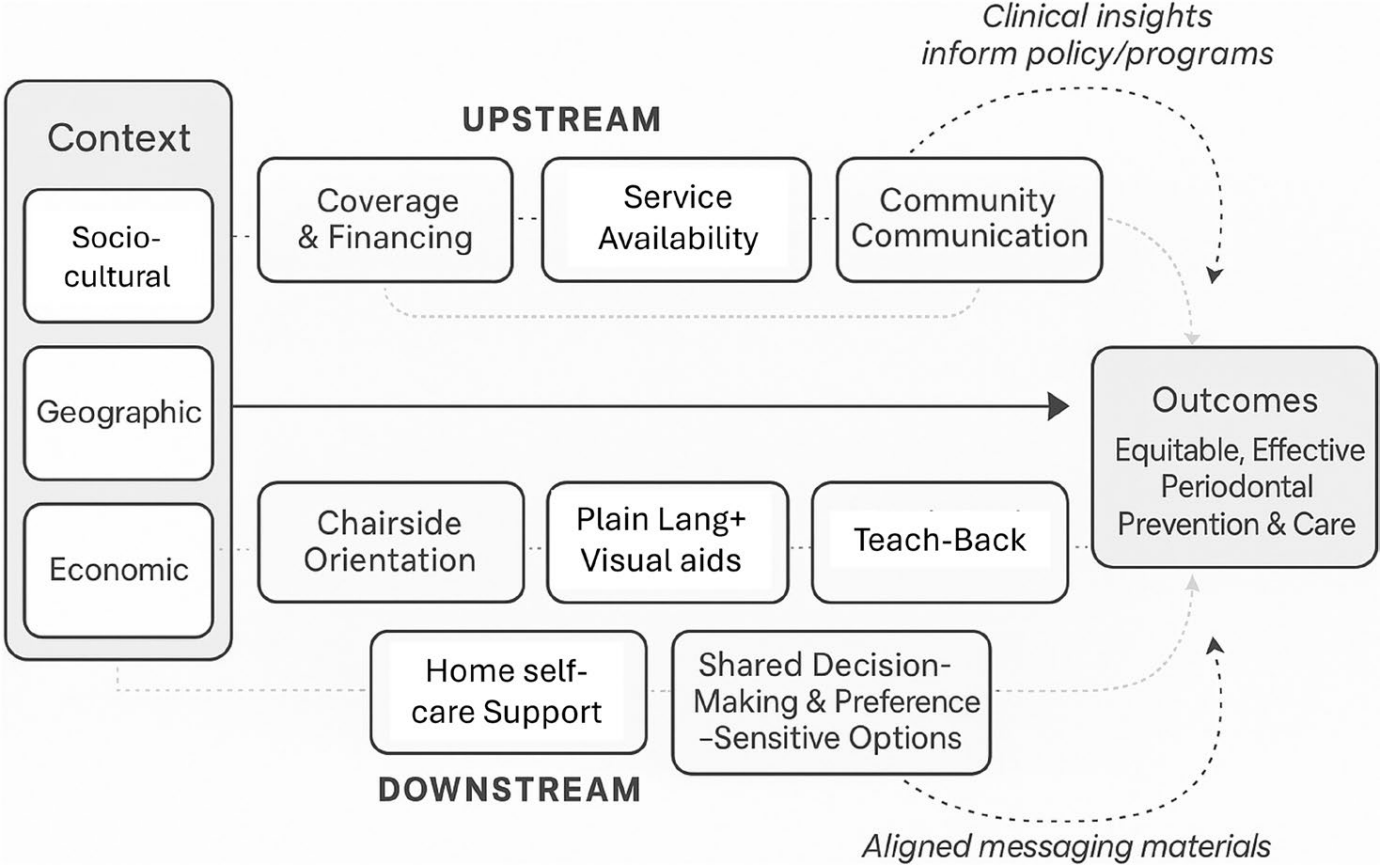
Integrated Upstream–Downstream Model for Periodontal Care. This model illustrates how equitable periodontal health depends on the interaction of upstream structural action and downstream patient‐centered care. Upstream measures—such as policies that reduce social and economic barriers and strengthen community communication—create the conditions for prevention. Downstream strategies translate these conditions into daily practice through supportive clinical encounters and sustained home self‐care support, free of patient‐blaming. Contextual factors—sociocultural, geographic, and economic—shape both levels, while feedback loops ensure that insights from clinical practice continually inform policy and community programs.

Building on the patient‐centered perspective outlined above, an effective periodontal strategy could align upstream determinants with downstream support, avoiding the pitfalls of patient‐blaming while acknowledging everyday constraints (Figure 3). Upstream components include affordable, timely access to periodontal care; sustained, co‐created communication at the community level; and policies that reduce time, cost, and transport barriers [164]. Downstream components occur chairside and at home: brief orientation to disease course and early signs, plain‐language education with visual aids, teach‐back to confirm understanding, and shared decision‐making that respects patient preferences and constraints [165]. Crucially, the two axes of this model are bidirectionally linked: upstream policies make recommended behaviors feasible, while downstream clinical encounters surface contextual barriers and preferences that can be fed back into community programs and policy. Parallels from other areas of medicine show that aligning structural action with patient‐centered practice is both feasible and impactful. In chronic pain, integrative, biopsychosocial approaches and ethnographic studies have reshaped routines and improved care processes by addressing social context and clinician–patient dynamics [166, 167]. In acute pain, attention to cultural framing has improved communication and reduced distress [168]. In infectious‐disease control and preparedness, anthropological engagement with communities—around care‐seeking, risk perception, and ritual practice—has increased the effectiveness and acceptance of public‐health interventions [169, 170].

This integrated approach is inherently contextual—what works in a rural setting with scarce providers will differ from an urban, uninsured population—and should therefore be tailored with local stakeholders (patients, community leaders, hygienists, physicians, and social workers) to ensure reach and fit [171].

Policy is necessary, not optional. Anthropologically informed clinical practice is necessary but not sufficient: durable impact requires policy—e.g., coverage for prevention and maintenance, integration of dentistry with primary care/UHC, reduced time and transport barriers, and investment in community health‐worker/cultural‐broker models [119, 120, 169, 170].

## 6 Conclusion: Toward Biocultural Periodontics

Periodontitis is shaped as much by social and cultural forces as by microbial dysbiosis. Distinguishing disease, illness, and sickness clarifies those layers and grounds a genuinely biopsychosocial model of care [53, 55]. Medical anthropology's insistence on distinguishing disease (the objective pathology), illness (the subjective experience), and sickness (the social dimension) provides a robust conceptual framework for understanding these interlocking layers [46], and we believe that distinguishing Periodontal Disease, Periodontal Illness and Periodontal Sickness is a useful heuristic tool to fully understand Periodontitis in all its aspects. Through the lens of EMs and semantic networks, everyday words, cultural memory, and moral judgment shape how a patient interprets bleeding gums, how promptly help is sought, and how clinical advice is received [51, 131]. Applying these anthropological perspectives can reveal how structural violence, socioeconomic disparities, and cultural differences impede early diagnosis and effective management [57]. As in chronic and acute pain services and in epidemic response—domains where medical anthropology has helped reconfigure models of care—the clinical shifts proposed here must be matched by political and policy commitments that reduce structural violence and ensure access; without these, their effects will be constrained [166, 167, 168, 169, 170].

Finally, bridging both the communication gap—ensuring that key messages reach people—and miscommunication—ensuring those messages fit their lived realities—is foundational to primordial and primary prevention in periodontitis. To close these gaps, care must be culturally attuned, draw upon the expertise of allied professionals, and extend into the communities where patients live [172]; only then can treatment transcend textbook protocols and acknowledge the realities of individuals who face financial constraints, enduring cultural orientations, and the burden of social stigma in their pursuit of oral health [139].

## Disclosure

AI Statement: This manuscript did not use artificial intelligence in any capacity.

## Conflicts of Interest

Elena Calciolari serves as an Editorial Board member of the Journal of Periodontal Research and is a co‐author of this article. In accordance with Wiley's standard policies for submissions by Editors, she was excluded from the editorial decision‐making related to this article and remained blinded throughout the peer‐review process.

## Data Availability

Data sharing not applicable to this article as no datasets were generated or analyzed during the current study.
